# Association between institutional affiliations of academic editors and authors in medical journals

**DOI:** 10.1093/eurpub/ckac129.465

**Published:** 2022-10-25

**Authors:** R Palladino, R Alfano, M Moccia, F Barone-Adesi, A Majeed, M Triassi, C Millett

**Affiliations:** 1 Public Health Policy Evaluation Unit, Imperial College, London, UK; 2 Department of Public Health, University, Naples, Italy; 3 CIRMIS, University, Naples, Italy; 4 Centre for Environmental Sciences, Hasselt University, Diepenbeek, Belgium; 5 MS Clinical Care and Research Centre, Department of Neuroscience, Naples, Italy; 6 Department of Translational Medicine, Università del Piemonte Orientale, Novara, Italy; 7 Research Center in Emergency and Disaster Medicine, Università del Piemonte Orientale, Novara, Italy; 8 Department of Primary Care and Public Health, Imperial College of London, London, UK

## Abstract

**Background:**

Most of the literature on conflict of interest (COI) has not focused on the role of academic editors and their possible COIs, although academic editors often hold senior faculty positions at universities, which might be considered a COI if this influences towards a more favourable processing to articles submitted by institutional colleagues. The current study aims to assess whether academic editor affiliation, a potential COI, can influence academic institution ranking as top contributor in the biomedical field.

**Methods:**

We conducted a cross-sectional analysis extracting publicly available data from the 2019 Clarivate InCites Journal Citation Reports for journals in the “Medicine, General & Internal” category and from each journal website. We constructed the following study outcomes: i) being a top 5 academic contributor for the peer-review journal of interest (yes/no), ii) being a top 10 academic contributor for the peer-review journal of interest (yes/no), and iii) ranking position as top 50 academic contributor for the peer-review journal of interest. Mixed-effect linear and logistic regression models were employed, as appropriate.

**Results:**

We included 114 journals in our analysis, 49% were open-access only. Sharing same affiliation of any of the editorial board members was associated with a 6.7 and 5.6 greater likelihood of being top 5 and top 10 contributors, respectively (95%CI 5.07-8.73 and 4.34-7.22). Similarly, sharing same affiliation was associated with being 12.1 places higher as top contributor (95%CI 10.35-13.81). When considering the editor in chief affiliation solely, association was even stronger.

**Conclusions:**

We found that academic editors sharing the same institutional affiliation with authors was strongly associated with the likelihood of that institution of being a top contributor. Shared institutional affiliations between editors and authors should be clearly stated as part of an open and transparent peer-review process.

**Key messages:**

• Editors sharing same affiliation with authors was strongly associated with the likelihood for the institution the editor was affiliated with of being top contributor for academic medical journals.

• Shared institutional affiliations between editors and authors should be clearly stated as part of an open and transparent peer-review process.

